# FINANCIAL DECISION MAKING AND WEALTH LOSS IN OLDER ADULTS WITH EARLY MEMORY LOSS: THE WALLET STUDY

**DOI:** 10.1093/geroni/igad104.3038

**Published:** 2023-12-21

**Authors:** Peter Lichtenberg, Emily Flores, Vanessa Rorai

**Affiliations:** Wayne State University, Detroit, Michigan, United States; Wayne State University, Detroit, Michigan, United States; Healthier Black Elders Center, Detroit, Michigan, United States

## Abstract

Angisani and Lee (2019) used the HRS to determine that wealth loss was related to early memory loss. Using methods described elsewhere (Lichtenberg et al., 2022), we investigated personal finance behaviors and wealth loss. using 12 months of checking account statements and follow up interviews with 114 older adults; 67 of whom had self-reported cognitive impairment (CI), and two-thirds of who were African American. Participants were largely recruited from research registries with the Michigan ADRC and the Michigan Center for Urban African American Aging Research. Mean age was 72 years mean education was 15 years, and 70% of the sample were women. There were no differences between the group on demographic variables, financial literacy (3-item Lusardi and Mitchell scale) or annual income. Bivariate analyses (t tests) indicated that those with CI were significantly more likely to suffer wealth loss across the 12 month period. Those with CI had significantly poorer scores on a person-centered financial decision making scale, and reported significantly lower IADL skills. In addition, those with CI were more likely to financially helping others on a regular basis, and reported a higher rate of financial exploitation. Correlations between wealth loss and CI (r=.22; p<.05), decision-making (r=.54; p<.05) and helping others on a regular basis (r=.51; p<.05). A logistic regression predicting wealth loss resulted in two measures significantly predicting wealth loss; financial decision-making and helping someone else financially on a regular basis.

Conclusions: (1) early CI is related to wealth loss and (2) decision-making intersects with wealth loss.

